# Parental Knowledge, Attitudes and Practices on Antibiotic Use in Children: A Cross-Sectional Study from Tbilisi, Georgia

**DOI:** 10.3390/antibiotics15030260

**Published:** 2026-03-02

**Authors:** Tata Imnadze, Nana Mebonia, Lile Malania, Ekaterine Ruadze

**Affiliations:** 1 National Center for Disease Control and Public Health (NCDC), 0198 Tbilisi, Georgia; 2 Mediterranean and Black Sea Programme in Intervention Epidemiology Training (MediPIET), European Centre for Disease Prevention and Control (ECDC), 171 83 Stockholm, Sweden; 3 Faculty of Medicine, Public Health and Epidemiology Department, Tbilisi State Medical University, 0186 Tbilisi, Georgia

**Keywords:** drug resistance, microbial, antimicrobial stewardship, health knowledge, attitudes, practice, antibiotic misuse, Georgia (the country)

## Abstract

**Background/Objectives**: Inappropriate antibiotic use in children is a major driver of antimicrobial resistance (AMR), especially in low- and middle-income countries. In Georgia, little is known about parental behaviors related to pediatric antibiotic use. This cross-sectional study assessed knowledge, attitudes, and practices (KAP) related to antibiotic use and AMR among parents of preschoolers in Tbilisi kindergartens, identifying factors associated with inappropriate practices to inform stewardship interventions. **Methods**: During March–May 2025, we conducted a two-stage cluster-sampled cross-sectional survey of parents of children aged 2–6 years attending public kindergartens in Tbilisi, Georgia. We assessed knowledge, attitudes, and practices concerning antibiotics and AMR. Multivariable Poisson regression was utilized to identify factors associated with inappropriate antibiotic use, defined as any of the following: early discontinuation, use without a prescription, or saving leftovers. **Results**: In total, 64.3% (95% CI 60.3–68.1%) reported antibiotic use in the past year, with 27.9% of respondents engaging in ≥1 inappropriate practice. Common reasons for use included bronchitis (35.9%) and sore throat (20.0%); Most antibiotics were prescribed by physicians (77.5%) or administered directly in a clinical setting (16.8%); 18.6% were obtained without a prescription. Forty percent believed antibiotics kill viruses. High knowledge (aPR 0.66, 95% CI 0.45–0.97) was independently associated with lower inappropriate practice; other sociodemographic factors were not significantly associated. **Conclusions**: Inappropriate pediatric antibiotic use in Tbilisi remains widespread and is linked to parental knowledge gaps rather than demographic factors. Interventions should prioritize correcting key misconceptions among caregivers, while addressing prescribing practices within outpatient settings. Strengthening both public and provider awareness may be essential for effective AMR containment in Georgia.

## 1. Introduction

Antimicrobial resistance (AMR) is a major global health threat. It was estimated to be associated with 4.95 million deaths in 2019, including 1.27 million deaths directly attributable to resistant infections [1]. Inappropriate antibiotic use is a key driver, especially in outpatient and community settings where regulatory oversight is weak and diagnostic support is limited [2,3]. In children, antibiotics are frequently prescribed for conditions such as viral upper respiratory tract infections, despite clear clinical guidelines advising against their use in these self-limiting, non-bacterial illnesses [4,5,6]. In several settings, more than one in five outpatient visits result in an antibiotic prescription, and early and repeated exposure in childhood contributes to the emergence and spread of AMR [7,8].

Misuse is influenced by limited public awareness, sociocultural expectations, and gaps in pharmaceutical regulation. Practices such as obtaining antibiotics without prescription, stopping treatment early, and using antibiotics without diagnostic confirmation contribute to resistance and pose individual risks for children [9,10,11]. Studies from low- and middle-income countries show that caregivers often have limited knowledge about antibiotics and AMR, hold misconceptions about when antibiotics are appropriate, and may self-medicate or pressure clinicians to prescribe [10,12,13,14,15]. Structural and social determinants, including maternal education, household income, and access to healthcare, further influence these practices [16,17,18].

Children are particularly vulnerable: early-life antibiotic exposure can disrupt the developing microbiome and has been linked to long-term health consequences such as asthma, obesity, and immune dysregulation in both human and animal studies [19,20]. These risks make caregiver practices a critical target for stewardship, especially in settings where enforcement of prescription-only policies is inconsistent.

In Georgia, policy efforts to contain AMR have included the development of a national action plan and formal regulations prohibiting over-the-counter antibiotic sales [21,22]. However, enforcement remains inconsistent, and informal dispensing still occurs. Public knowledge about AMR is low [23], and no published studies have focused on caregivers of young children, a group that strongly influences household antibiotic use. Existing studies have primarily focused on adults, healthcare workers, or institutional settings [2]. Notably, a point prevalence study (PPS) conducted between 2015 and 2019 in Georgian pediatric hospitals revealed widespread empirical antibiotic prescribing and limited microbiological testing, highlighting broader systemic challenges in antimicrobial stewardship—even within the formal healthcare system [24].

Parents of children aged 2–6 years represent a particularly relevant population for antimicrobial stewardship research. Preschool children frequently present with respiratory infections in outpatient settings [25], where antibiotics are commonly prescribed, placing caregivers at the center of antibiotic-related decision-making within the household. Public kindergartens provide a structured and geographically distributed sampling frame, enabling cluster-based recruitment of caregivers and enhancing population-level representation across Tbilisi.

To address this gap, we conducted a cross-sectional study to assess the knowledge, attitudes, and practices (KAP) related to antibiotic use and AMR among parents of young children attending public kindergartens in Tbilisi, Georgia. We also aimed to identify sociodemographic and KAP-related factors associated with inappropriate antibiotic practice, with the goal of informing targeted interventions and contributing to Georgia’s broader AMR containment strategy.

## 2. Results

### 2.1. Participant Characteristics

A total of 639 parents participated (response rate 85.2%). Most respondents were women (93.4%), and the majority were aged 25–39 years (71.8%). Nearly half held a bachelor’s degree (47.2%). Weighted characteristics closely matched the unweighted sample distribution (Table 1).

### 2.2. Antibiotic Use in Children

Overall, 64.3% of parents (*n* = 412, 95% CI 60.3–68.1) reported giving their child antibiotics in the past 12 months. Among parents of 2–3-year-olds, the reported prevalence was (68.9%, 95% CI 63.2–74.6).

Among those who administered antibiotics, most reported 1–2 treatment courses in the past year (78.6%, 95% CI 73.7–83.4). Smaller proportions reported 3–5 courses (11.5%, 95% CI 7.7–15.3) or more than 5 courses (3.1%, 95% CI 1.5–4.6).

Among antibiotic users in the past year, 27.9% (*n* = 114, 95% CI 23.3–32.9) engaged in at least one caregiver-controlled inappropriate practice (early discontinuation, use without a prescription, or saving leftovers).

### 2.3. Reasons for Antibiotic Use

**Figure 1:**
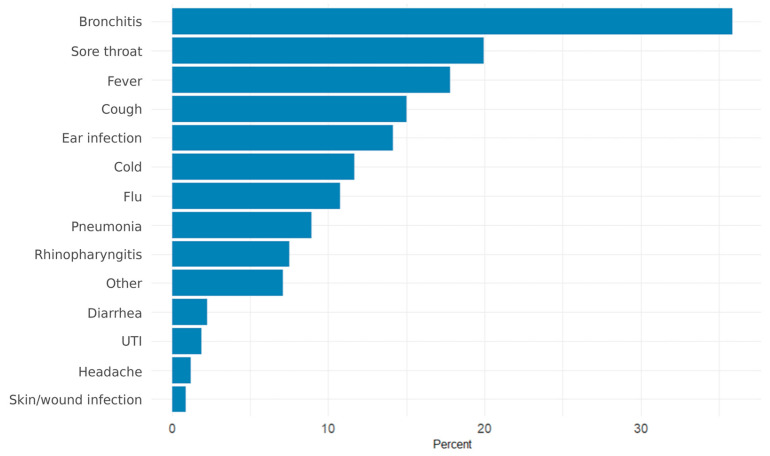
Reported reasons for the child’s most recent antibiotic course among parents who administered antibiotics in the past 12 months (*n* = 412). Bars represent weighted percentages. Multiple responses were permitted.

Among parents or guardians who had sought antibiotic treatment for their children, the most frequent reasons were bronchitis (35.9%, 95% CI 31.7–40.1), sore throat (20.0%), fever (17.8%), cough (15.0%), ear infections (14.2%), and colds (11.7%) (Figure 1).

### 2.4. Sources and Administration of Antibiotics

Most antibiotics were given following a medical prescription (77.5%, 95% CI 72.8–82.2). Administration by a practitioner was reported by 16.8% (95% CI 12.6–21.0). Use based on prior experience (3.8%, 95% CI 1.8–5.8) or the advice of a pharmacist (0.4%) was uncommon.

Non-prescription acquisition was reported by 18.6% (95% CI 14.1–22.6) of parents. Pharmacies remained the primary source of antibiotics overall, with 62.3% (95% CI 56.8–67.8) of parents obtaining the most recent course with a prescription.

Diagnostic testing before treatment was reported by 79.2% (95% CI 74.0–84.3), while 20.6% (95% CI 15.4–25.8) reported no testing.

### 2.5. Knowledge About Antibiotics and AMR

Knowledge levels varied across topics. About four in ten parents believed antibiotics kill viruses (39.7%, 95% CI 34.6–44.7), while a similar proportion correctly disagreed. Over half (57.7%, 95% CI 52.4–63.0) knew that antibiotics do not help against influenza-like illness.

Most parents recognized that unnecessary antibiotic use can reduce effectiveness (74.0%, 95% CI 69.8–78.2).

Awareness of side effects was moderate: 60.9% (95% CI 56.3–65.6) knew antibiotics may cause adverse effects such as diarrhea.

The mean knowledge score was 2.3 (SD 1.3) out of 4, with a median of 2 (IQR 1–3) (Appendix A, Table A1). Overall, 10.0% of parents (95% CI 7.5–12.5) answered none of the knowledge items correctly, 18.2% (95% CI 14.6–21.9) scored one, 25.6% (95% CI 21.9–29.4) scored two, 22.3% (95% CI 18.5–26.1) scored three, and 23.8% (95% CI 19.1–28.5) achieved full scores. These findings indicate moderate overall knowledge with considerable heterogeneity and no clear clustering at the extremes.

### 2.6. Attitudes Toward Antibiotic Use

The majority of parents reported that they would consult a doctor before giving antibiotics to their child (87.2%, 95% CI 83.1–91.3).

Desire for antibiotic prescriptions was mixed: 57.4% (95% CI 52.7–62.1) did not desire the doctor to prescribe antibiotics when their child was ill, while 25.6% (95% CI 21.0–30.1) somewhat desired one, and 10.1% (95% CI 7.0–13.3) strongly desired one.

The mean attitude score was 1.47 (SD 0.62), with a median of 2 (IQR 1–2). A total of 6.2% of parents (95% CI 3.6–8.8) had the lowest attitude score, 39.4% (95% CI 34.9–43.9) had intermediate scores, and 52.4% (95% CI 47.3–57.6) had the highest attitude score.

### 2.7. Reported Practices

Completion of prescribed treatment was common: 70.4% (95% CI 66.4–74.4) reported always completing the course. Early discontinuation occurred in 14.7% (95% CI 11.4–17.9).

Among those who stopped treatment early, most did so because the child improved (67.6%, 95% CI 54.7–80.5). Side effects (21.7%) and child refusal (9.5%) were less frequent reasons.

Among parents who had given antibiotics to their children (*n* = 412), practice scores showed that most did not report any inappropriate practices. The mean practice score was 0.33 (SD 0.58), median 0 (IQR 0–1). Appropriate practice (score = 0) was observed in 72.1% (95% CI 67.3–76.9), while 22.4% (95% CI 17.8–27.1) reported one inappropriate practice, 5.3% (95% CI 3.1–7.5) reported two, and <1% reported three.

Reported actions linked to inappropriate practice are summarized in Table 2. Component behaviors included non-prescription acquisition (18.6%), early discontinuation (14.7%), and storing leftover antibiotics for later use (8.1%).

### 2.8. Information Sources and Perceived Impact

One-third of parents (34.2%, 95% CI 30.0–38.4) reported receiving information about antibiotics or AMR in the past year. Among them, 74.5% (95% CI 68.6–80.4) stated that this information changed their views.

**Figure 2:**
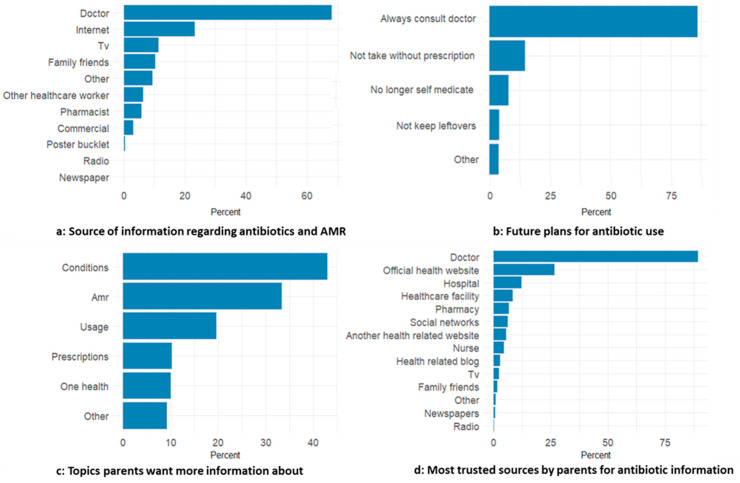
Sources of information, future practices, information needs, and trusted communication channels regarding antibiotics and AMR among parents in Tbilisi. (**a**) Sources of information received in the past year; (**b**) Intended future behaviors related to antibiotic use; (**c**) Topics for which parents indicated a desire for additional information; (**d**) Trusted sources of information.

As shown in Figure 2a, doctors were the most commonly reported information source, followed by the internet, while traditional media and interpersonal sources were less frequently reported. Figure 2b indicates that most parents intended to always consult a doctor, and a smaller proportion reported plans to avoid using antibiotics without a prescription. In Figure 2c, parents most often wanted more information on which conditions require antibiotics and on AMR, followed by safe use and prescription-related topics. Figure 2d shows that doctors were the most trusted source (89.4%, 95% CI 86.9–91.8), followed by official websites (26.7%). Trust in all other sources was below 12%.

### 2.9. Crude Associations with Inappropriate Antibiotic Practice

In crude regression analysis, several factors were associated with inappropriate antibiotic practice (Table 3).

Higher education, higher income, having two children, high knowledge, and positive attitudes were each associated with lower prevalence of inappropriate use.

Misconceptions showed the strongest associations: parents who believed antibiotics help for colds had more than twice the prevalence of inappropriate practice (PR 2.17, 95% CI 1.52–3.09), and those uncertain whether unnecessary antibiotic use leads to reduced effectiveness also reported higher misuse (PR 1.75, 95% CI 1.23–2.48). Misconceptions about cold treatment and uncertainty about AMR showed the largest relative differences in prevalence.

### 2.10. Multivariable Model

After restricting analyses to participants with complete data, multivariable models were fitted among parents who reported antibiotic use in the past year, accounting for clustering by kindergarten. Variance inflation factors indicated no concerning collinearity among predictors (all GVIF^(1/2df)^ < 1.2).

In the adjusted model (Appendix A, Table A2), only knowledge level remained significantly associated with inappropriate antibiotic practice. Parents with high knowledge scores had a lower adjusted prevalence of inappropriate use compared with those with low knowledge scores (aPR 0.66, 95% CI 0.45–0.97).

Number of children showed a borderline association: parents with two children had somewhat lower prevalence of inappropriate practice compared with those with one child, although this did not reach statistical significance (aPR 0.68, 95% CI 0.45–1.04).

Household income, parental education, and attitude were not significantly associated with inappropriate practice after adjustment (*p* > 0.05 for all).

## 3. Discussion

### 3.1. Summary of Main Findings

This study provides new evidence on antibiotic-related knowledge, attitudes, and practices among parents of young children in Tbilisi, Georgia. Antibiotic exposure in early childhood was common. Nearly two-thirds of parents reported administering antibiotics to their child in the past 12 months, with even higher use among parents of two- and three-year-olds. These levels exceed estimates reported for the general population in Georgia [23] and raise concern given the vulnerability of the developing childhood microbiome.

Despite generally moderate knowledge and positive attitudes, inappropriate caregiver-controlled practices persisted. About one in four parents who had used antibiotics reported at least one inappropriate behavior, most commonly non-prescription acquisition, followed by early discontinuation, or storage of leftovers. While appropriate behaviors were reported by many parents, the persistence of these practices highlights ongoing risks for antimicrobial resistance.

High antibiotic use among preschool-aged children is consistent with patterns reported in many settings where respiratory symptoms are frequent and diagnostic uncertainty is common. However, the magnitude observed in this study suggests that antibiotic exposure in Georgia remains high despite prescription-only regulations and clinical guidelines that discourage antibiotic use for viral infections.

Importantly, most antibiotics reported in this study were prescribed by healthcare professionals. This suggests that caregiver-controlled inappropriate practice is not driven solely by parental misunderstanding or non-prescription acquisition. Instead, it occurs within a wider care pathway in which most antibiotic courses are clinician-prescribed. We did not assess the appropriateness of clinician prescribing; however, contextual factors such as diagnostic uncertainty, limited diagnostic support, and parental expectations during acute illness episodes may influence antibiotic use. Similar patterns have been reported in other low- and middle-income countries in the WHO European Region, where legal restrictions coexist with high pediatric antibiotic use.

Non-prescription acquisition was reported despite formal regulations. This suggests an implementation gap between policy and practice rather than absence of regulation. Without consistent enforcement and accountability mechanisms, prescription-only laws alone are unlikely to substantially reduce inappropriate antibiotic use.

The most frequently reported indication for antibiotic use was bronchitis. This finding should be interpreted cautiously. In routine outpatient practice, diagnostic labels such as “bronchitis” may be applied broadly and may encompass a mix of viral and bacterial respiratory syndromes. Because diagnoses were parent-reported and not clinically validated, we cannot determine whether antibiotic prescribing was clinically appropriate for specific episodes. Diagnostic labeling may also shape parental expectations regarding antibiotic treatment, complicating efforts to distinguish caregiver behavior from provider prescribing norms.

Although a high proportion of parents reported that diagnostic testing preceded antibiotic use, this finding should be interpreted cautiously. The survey did not specify the type of test performed, and caregiver interpretations of “testing” may include physical examination, blood tests, or imaging rather than microbiological diagnostics. In outpatient pediatric care in Georgia, bacteriological testing and antimicrobial susceptibility testing are rarely performed prior to treatment, and prescribing is largely empirical. Therefore, reported testing should not be interpreted as evidence of guideline-concordant diagnostic stewardship.

Poor understanding or misconceptions about antibiotics and their use emerged as the strongest correlate of inappropriate practice. Parents with higher knowledge scores had a lower prevalence of inappropriate behaviors, while education, income, and attitudes were not independently associated after adjustment. This finding should not be interpreted causally. Antibiotic use is also influenced by prescribing and dispensing contexts, so knowledge alone may not fully determine behavior. The cross-sectional design cannot establish whether knowledge leads to better practice or whether parents who behave appropriately acquire more accurate information. Still, the association supports existing evidence that correcting specific misconceptions may be a practical intervention target. Our findings on caregiver knowledge should be interpreted within this broader context. Because our primary outcome combined several caregiver-controlled practices, determinants may differ by practice, and future analyses could model these components separately.

### 3.2. Strength and Limitations

Strengths include the cluster sampling approach, large, representative sample, high response rate, and a clearly defined outcome focused on caregiver-controlled behaviors. The use of GEE models appropriately accounts for clustering at the kindergarten level.

Several limitations must be considered in interpreting our findings.

Selection bias is possible, as we could not confirm strict adherence to the distribution protocol in kindergartens. More engaged or health-conscious parents may have been more likely to participate, potentially inflating knowledge scores or underestimating misuse. Despite follow-up with kindergarten managers, non-random participation remains a concern.

Social desirability bias may have also led to underreporting of sensitive practices, such as self-medication or early treatment discontinuation. If present, such biases likely result in conservative estimates meaning actual rates of misuse may be even higher.

Recall bias is inherent in self-reported data, especially over a 12-month reference period. Some parents may have misclassified symptom episodes or antibiotic courses. We attempted to mitigate this by focusing on the past year, but inaccuracies remain. Non-response bias is another consideration: some kindergartens had incomplete returns, and non-responders may systematically differ from participants. While we used post-stratification weights to adjust for this, residual bias may persist.

Measurement limitations also apply. The binary KAP scoring system may oversimplify complex decision-making and reduce variability by collapsing potentially meaningful gradients in knowledge and attitudes. In addition, dichotomization the knowledge score may have reduced detail and limited detection of more nuanced associations. Treating “don’t know” responses as incorrect may conflate uncertainty with misinformation. In the practice score, all inappropriate behaviors were weighted equally despite differing clinical implications.

Finally, survey terminology may have been interpreted inconsistently. “Diagnostic testing” may have referred to anything from a physical examination to laboratory tests. Reported indications should therefore be interpreted as caregiver perceptions rather than verified clinical diagnoses. Diagnostic labels such as “bronchitis” may be used broadly in routine practice and may represent heterogeneous syndromes, including viral and bacterial conditions. This limits inference about appropriateness by indication. Similarly, “administered by a healthcare provider” might have been read as “prescribed by” rather than literal administration especially given the focus on oral medications. Additionally, high reported frequencies of antibiotic use (e.g., >5 times/year) among healthy children suggest misunderstanding of what constitutes a treatment course. This may have led to overestimation of true antibiotic exposure.

## 4. Materials and Methods

### 4.1. Study Design and Setting

We conducted a cross-sectional survey among parents of children aged 2–6 years enrolled in public kindergartens in Tbilisi, Georgia. Data collection took place between March and May 2025. The study aimed to measure parental KAP regarding antibiotic use and antimicrobial resistance (AMR).

In Georgia, public kindergartens are municipal early childhood education settings that enroll preschool-aged children, including children aged 2–6 years.

### 4.2. Study Population

Eligible participants were parents aged 18 years or older who had a child attending one of the selected public kindergartens; one parent per child was invited to participate. Parents unable to read Georgian were excluded. Participation was voluntary, and returning a completed questionnaire implied informed consent.

### 4.3. Sample Size Estimation and Sampling Strategy

The minimum required sample size was calculated using a single proportion formula, assuming a 50% prevalence of inappropriate antibiotic practice, 5% margin of error, and 95% confidence level. This yielded 385 participants. Because the study used a cluster sampling design, we applied a design effect of 1.6, informed by WHO guidance for complex survey designs and selected as a conservative value [26], resulting in 616 participants. To account for an anticipated 20% non-response rate, the target sample size was increased and rounded up to 750 parents.

We used a two-stage cluster sampling design. In stage one, 50 kindergartens were selected from all 190 public kindergartens in Tbilisi, using probability proportional to size (PPS) based on total child enrollment. In the second stage, a fixed number of 15 questionnaires per selected kindergarten were distributed to parents. To capture potential variation in antibiotic use across child age groups, each kindergarten was assigned a specific age group (2–5 years) for targeted distribution.

The PPS design combined with a fixed number of questionnaires per kindergarten produced a mathematically self-weighting sample. Non-response weights were calculated as the inverse of the kindergarten-specific response rate. These weights were normalized to a mean of 1.0 for variance estimation.

### 4.4. Instruments and Data Collection

A structured, paper-based questionnaire was adapted from the WHO European Region knowledge, attitudes and behaviors survey on antibiotic use and AMR (2022) [23], with modifications for relevance to caregivers of young children in Georgia. Additional items were based on publicly available KAP surveys. The adapted Georgian version was reviewed for clarity and cultural appropriateness by the study team, with consultation from a practicing pediatrician and AMR/public health specialist. The instrument was pilot-tested before field implementation.

Questionnaires were distributed via kindergarten staff to eligible parents and returned anonymously. Staff were instructed that participation was voluntary and that they should not track which parents responded.

### 4.5. Measures and Variable Definitions

Sociodemographic information included parent age, sex, education, employment status, household income, and number of children (Details on category collapsing and variable recoding are provided in Appendix B, Section Category Collapsing and Variable Recoding Strategy).

The primary outcome was inappropriate antibiotic practice. This captured caregiver-controlled behaviors inconsistent with recommended stewardship, specifically: obtaining antibiotics without a prescription, stopping a prescribed course prematurely, or storing antibiotics for future use. Each inappropriate practice (including “don’t know”) was given a score of 1, while appropriate practice was scored as 0. A binary outcome classified respondents as having appropriate practice (score = 0) or inappropriate practice (score ≥ 1) if they reported at least one of the three inappropriate behaviors. Provider-driven factors, such as antibiotics prescribed for viral illnesses or absence of diagnostic testing, were not included in the definition because these decisions fall outside parental control.

Knowledge about antibiotics and antimicrobial resistance was measured using four factual items assessing whether respondents understood that antibiotics do not kill viruses, are not effective for colds, that unnecessary antibiotic use reduces its effectiveness, and that antibiotics may cause side effects, such as diarrhea. Each correct answer was given a score of 1, while incorrect and “don’t know” responses were scored as 0. “Don’t know” responses were grouped with incorrect answers because both indicate absence of correct knowledge relevant to antibiotic use. A total Knowledge Score (range 0–4) was calculated and dichotomized into high knowledge (≥3 correct responses) and low knowledge (≤2 correct responses). Dichotomization was used to improve interpretability and to limit model complexity given events-per-variable considerations in the multivariable analysis.

Parental attitudes toward antibiotic use were measured using two items that assessed the desire for antibiotic prescription from a doctor when a child is ill and the likelihood of consulting a doctor before giving antibiotics. Responses indicating appropriate attitudes (not desiring an antibiotic prescription and intending to consult a doctor before giving antibiotics to their child) were scored as 1. The resulting Attitude Score ranged from 0–2 and was categorized as positive (score of 2) or negative (score of 0–1).

### 4.6. Data Entry and Management

Completed questionnaires were entered into KoboToolbox (server version 2.025.x) and exported to R (version 4.3.1). Data entry and cleaning occurred concurrently. Logical checks, skip pattern verification, and cross-validation with original questionnaires were performed to minimize errors. No personal identifiers were collected.

### 4.7. Statistical Analysis

All analyses were conducted in R (version 4.3.1). Data were first described using weighted proportions and 95% confidence intervals, applying normalized non-response weights. Weighted estimates were obtained with the survey R-package (version 4.4-2), specifying kindergarten as the primary sampling unit and using Taylor linearization for variance estimation.

For analytical models, we used generalized estimating equations (GEE) with a Poisson distribution [27] and log link to estimate adjusted prevalence ratios (PR) and 95% confidence intervals for the common outcome. Kindergarten was specified as the clustering variable, with an independence working correlation structure. Robust (sandwich) standard errors were used to account for within-cluster correlation.

Covariates for the multivariable model were selected based on conceptual relevance, evidence of association in crude analyses (95% confidence intervals excluding the null value), and assessment of multicollinearity using generalized VIF. Multicollinearity was assessed across all candidate predictors considered for the multivariable model; no concerning collinearity was observed. The number of predictors was constrained according to events-per-variable considerations to minimize overfitting. The final model included parental education, socio-professional category, household income, number of children, knowledge level, and attitude. Number of antibiotic courses was not included due to evidence of recall error and implausible values. Sex and age of parents were excluded because they showed no association in crude analyses. As sensitivity analyses, we fitted survey-weighted and unweighted Poisson models with a log link and unweighted logistic regression models. These produced similar patterns of association and did not change the substantive conclusions, so we report unweighted GEE Poisson results as the primary analysis.

### 4.8. Ethical Considerations

Ethical approval was obtained from the Institutional Review Board of the National Center for Disease Control and Public Health (NCDC), Georgia (IRB #2022-085). The questionnaire was anonymous and no identifiable information was collected.

### 4.9. Generative AI Disclosure

Generative artificial intelligence tools (ChatGPT by OpenAI) were used to assist with wording refinement, and structural editing. No AI tools were used for data collection, analysis, or interpretation. All statistical analyses were performed by the authors.

## 5. Conclusions

This study provides the first targeted assessment of antibiotic-related knowledge, attitudes, and practices among parents of young children in Georgia. The findings highlighted a disconnect between recommended antibiotic stewardship practices and real-world antibiotic use, shaped by both caregiver behavior and provider prescribing practices. A substantial proportion of parents reported inappropriate antibiotic use, indicating that misuse remains normalized across social and educational groups rather than confined to specific subpopulations. Knowledge deficits emerged as the most consistent correlate of inappropriate practices, while demographic characteristics showed limited explanatory value.

These findings suggest that efforts to improve antibiotic stewardship in Georgia must extend beyond general awareness raising. Targeted correction of common misconceptions, particularly regarding antibiotic use for viral illnesses, early discontinuation, and reuse of leftovers, remains necessary. Given that healthcare professionals were identified as the most trusted information source, stewardship messaging is likely to be most effective when integrated into routine maternal and child health services, vaccination visits, and pediatric consultations.

At the same time, improving parental knowledge alone will be insufficient. Most antibiotics reported in this study were prescribed by clinicians, indicating important opportunities to strengthen outpatient antimicrobial stewardship. Because prescribing appropriateness was not measured, our outcome reflects caregiver-controlled behaviors rather than clinician decision-making. Strengthening guideline-concordant prescribing, enhancing diagnostic support, and reinforcing stewardship practices in ambulatory pediatric care are critical priorities.

Regulatory enforcement also requires attention. Reported non-prescription access to antibiotics suggests fragmented implementation of prescription-only policies. Strengthening verification mechanisms, pharmacy oversight, and monitoring systems is essential to ensure that existing regulations translate into practice.

Overall, these findings provide a baseline for monitoring antibiotic-related behaviors among parents of young children in Tbilisi and for evaluating future interventions under Georgia’s national antimicrobial resistance strategy. Coordinated action across education, clinical practice, and regulation will be required to close the gap between policy, knowledge, and practice.

## Figures and Tables

**Table 1:** Demographic Characteristics of Survey Respondents (Unweighted and Weighted).

Characteristic	Unweighted ^1^	Weighted (Normalized) ^1^
	N = 639	N = 639
Gender		
Female	598 (93.6%)	597 (93.4%)
Male	41 (6.4%)	42 (6.6%)
Age group (years)		
18–24	30 (4.7%)	32 (5.0%)
25–39	457 (71.6%)	453 (71.0%)
40–54	146 (22.9%)	149 (23.3%)
55+	5 (0.8%)	5 (0.7%)
Unknown	1	
Education level		
No formal education	10 (1.6%)	9 (1.5%)
Low education (grades 1–8)	4 (0.6%)	5 (0.7%)
School (grades 9–12)	167 (26.8%)	168 (26.9%)
Bachelor	294 (47.2%)	294 (47.2%)
Masters	134 (21.5%)	133 (21.4%)
PhD	14 (2.2%)	14 (2.3%)
Unknown	16	16
Average monthly income (in Georgian lari)		
<500 (Below poverty)	16 (2.8%)	17 (3.1%)
500–999 (Low)	88 (15.5%)	90 (15.9%)
1000–1999 (Lower-middle)	211 (37.1%)	213 (37.5%)
2000–4999 (Upper-middle)	209 (36.8%)	204 (36.0%)
5000+ (High)	44 (7.7%)	43 (7.5%)
Unknown	71	71
Socio-professional category		
Unemployed	159 (25.4%)	156 (25.0%)
Manual-worker	4 (0.6%)	4 (0.7%)
Medical-worker	66 (10.6%)	68 (10.8%)
Office-worker	136 (21.8%)	135 (21.5%)
Other	99 (15.8%)	102 (16.3%)
Self-employed	148 (23.7%)	148 (23.6%)
Student	13 (2.1%)	13 (2.1%)
Unknown	14	14
Number of Children		
1	171 (26.8%)	167 (26.2%)
2	307 (48.1%)	308 (48.4%)
3	127 (19.9%)	128 (20.1%)
4	19 (3.0%)	17 (2.7%)
5	14 (2.2%)	16 (2.6%)
Unknown	1	1
Child’s age		
2	166 (26.0%)	167 (26.2%)
3	168 (26.3%)	168 (26.2%)
4	147 (23.0%)	142 (22.2%)
5	141 (22.1%)	146 (22.9%)
6	17 (2.7%)	16 (2.6%)

^1^ *n* (%). Unweighted counts and percentages reflect the observed sample. Unknown values indicate missing responses and are shown as counts only. Weighted estimates account for cluster sampling design and kindergarten-level non-response.

**Table 2:** Reported Antibiotic Practices.

Practice Item	Response Category	*n*	%	95% CI
Completion of antibiotic course (last used)	Completed full course	450	70.4	66.4–74.4
	Stopped early	94	14.7	11.4–17.9
	Never used antibiotics	94	14.7	11.1–18.3
Reasons for stopping early (among early stoppers)	Child improved	65	67.6	54.7–80.5
	Side effects	20	21.7	12.1–31.3
	Child refusal	9	9.5	3.8–15.1
	Forgot	2	2.0	–0.9–5.0
Source of last course of antibiotics	Pharmacy with prescription	256	62.3	56.8–67.8
	Pharmacy without prescription	76	18.6	14.1–22.6
	Administered by practitioner	69	16.8	12.6–21.0
	Other (leftovers/informal)	<5	<1	—
Storing leftover antibiotics for future use	Yes	49	8.1	5.7–10.5
	No	584	90.9	88.3–93.4
Diagnostic testing before giving antibiotics	Performed	323	79.2	74.0–84.3
	Not performed	88	20.6	15.4–25.8

All percentages are weighted. Completion of course and source of antibiotics use all parents as denominator; reasons for stopping early use only those who stopped early; practice score distribution uses only antibiotic users as denominator.

**Table 3:** Crude Associations Between Parental Characteristics and Inappropriate Antibiotic Practice.

Variable	Category	*n* with Inappropriate Use (% *)	PR	95% CI
Sex	Female (ref)	109 (18.2)	1	—
	Male	5 (12.2)	0.74	0.37–1.50
Age group (years)	18–24 (ref)	5 (16.7)	1	—
	25–39	82 (17.9)	1.14	0.50–2.61
	40+	26 (17.2)	1.26	0.55–2.86
Child’s age	2 years (ref)	36 (21.7)	1	—
	3 years	30 (17.9)	0.89	0.52–1.50
	4 years	20 (13.6)	0.78	0.51–1.20
	5 years	26 (18.4)	1.01	0.64–1.60
Education	School or below (ref)	50 (25.4)	1	—
	High education	64 (14.5)	0.62	0.44–0.86
Socio-professional category	Unemployed/student (ref)	27 (15.7%)	1	—
	Health-related	9 (13.6%)	0.7	0.36–1.38
	Office/professional	24 (17.6%)	1.09	0.72–1.65
	Other/manual-work/self-employed	52 (20.7%)	1.21	0.8–1.84
Income level	<2000 GEL (ref)	64 (20.3)	1	—
	≥2000 GEL	35 (13.8)	0.68	0.50–0.92
Number of children	1 (ref)	40 (23.4)	1	—
	2	38 (12.4)	0.61	0.40–0.92
	≥3	33 (22.6)	1.09	0.77–1.52
Knowledge level	Low (ref)	76 (21.8)	1	—
	High	38 (13.1)	0.6	0.41–0.87
Attitude	Negative (ref)	71 (24.3)	1	—
	Positive	43 (12.8)	0.68	0.50–0.94
Number of antibiotic courses (past year)	1–2 (ref)	81 (24.5)	1	—
	3–5	15 (31.9)	1.28	0.83–1.95
	Don’t remember	11 (36.7)	1.83	1.17–2.88
	>5	4 (28.6)	1.14	0.48–2.70
Belief: antibiotics kill viruses	False (ref)	33 (13.4)	1	—
	Don’t know	15 (15.6)	1.3	0.78–2.16
	True	61 (24.1)	1.55	0.99–2.42
Belief: antibiotics help colds	False (ref)	45 (12.2)	1	—
	Don’t know	14 (17.3)	1.48	0.96–2.29
	True	46 (33.1)	2.17	1.52–3.09
Belief: unnecessary use makes antibiotics ineffective	True (ref)	74 (15.9)	1	—
	Don’t know	25 (28.1)	1.75	1.23–2.48
	False	6 (15.4)	1.06	0.53–2.11
Belief: antibiotics cause side effects	True (ref)	72 (18.8)	1	—
	Don’t know	24 (15.5)	0.9	0.63–1.28
	False	9 (26.5)	1.41	0.84–2.35

* Row percentages: % of respondents in each category with inappropriate antibiotic use. Crude associations restricted to parents reporting antibiotic use in the past 12 months (*n* = 412). PR = prevalence ratio; CI = confidence interval. Categories with zero events, structural missingness, or NA-only responses removed for clarity.

## Data Availability

De-identified data and analysis code may be made available upon reasonable request to the corresponding author, subject to approval by the National Center for Disease Control and Public Health (NCDC) Institutional Review Board.

## Abbreviations

The following abbreviations are used in this manuscript:

AMR	Antimicrobial resistance
KAP	Knowledge, attitudes, and practices
PPS	Point prevalence study
WHO	World Health Organization
LMICs	Low- and middle-income countries
CI	Confidence interval
PR	Prevalence ratio
aPR	Adjusted prevalence ratio
GEE	Generalized estimating equations
GVIF	Generalized variance inflation factor
SD	Standard deviation
IQR	Interquartile range
IRB	Institutional Review Board
NCDC	National Center for Disease Control and Public Health
GEL	Georgian Lari

## Appendix A

**Table A1:** Distribution of knowledge, attitude, and practice (KAP) scores among parents of young children in Tbilisi, Georgia.

Score Category	Score	*n*	Weighted %	95% CI
Practice score (among antibiotic users; *n* = 412)	0	298	72.1%	67.3–76.9
	1	92	22.4%	17.8–27.1
	2	21	5.3%	3.1–7.5
	3	1	0.2%	—
Knowledge score (*n* = 639)	0	69	10.0%	7.5–12.5
	1	115	18.2%	14.6–21.9
	2	165	25.6%	21.9–29.4
	3	142	22.3%	18.5–26.1
	4	148	23.8%	19.1–28.5
Attitude score (*n* = 627)	0	41	6.2%	3.6–8.8
	1	251	39.4%	34.9–43.9
	2	335	52.4%	47.3–57.6

**Table A2:** Adjusted Associations Between Parental Characteristics and Inappropriate practice.

Parameter	Reference Level	Characteristic	aPR	95% CI
Socio Professional Category	Unemployed/student	Health-related	0.7	0.33–1.51
		Office/professional	1.5	0.88–2.55
		Other/manual-worker/self-employed	1.17	0.76–1.78
Education	School or below	High education	0.79	0.53–1.16
Income level	<2000 GEL	≥2000 GEL	0.8	0.54–1.18
Number of children	1	2	0.68	0.45–1.04
		≥3	1.04	0.75–1.46
Knowledge	Low	High	0.66	0.45–0.97
Attitude	Negative	Positive	0.89	0.62–1.3

## Appendix B

### Category Collapsing and Variable Recoding Strategy

Several categorical variables were collapsed prior to analysis to reduce sparse cells, improve statistical stability, and enhance interpretability of regression models. Collapsing decisions were defined a priori based on variable distributions and conceptual relevance.

Education was dichotomized into School or below and High education (bachelor’s degree or higher) to avoid sparsity in advanced degree categories. Parental age categories 40–54 years and ≥55 years were combined into a single 40+ group due to small numbers. Household income was dichotomized as Below 2000 GEL versus Above 2000 GEL, reflecting a meaningful socioeconomic threshold in the Georgian context. Number of children was grouped as 1, 2, and More than 2 to preserve family-size contrasts while maintaining adequate cell sizes. Employment status was collapsed into Employed and Not employed (including unemployed and students) to capture labor market participation rather than heterogeneous occupations.
